# Long COVID Syndrome Prevalence in 2025 in an Integral Healthcare Consortium in the Metropolitan Area of Barcelona: Persistent and Transient Symptoms

**DOI:** 10.3390/vaccines13090905

**Published:** 2025-08-26

**Authors:** Antonio Arévalo-Genicio, Mª Carmen García-Arqué, Marta Gragea-Nocete, Maria Llistosella, Vanessa Moro-Casasola, Cristina Pérez-Díaz, Anna Puigdellívol-Sánchez, Ramon Roca-Puig

**Affiliations:** 1 Primary Health Care, CAP Dr. Joan Planas, Consorci Sanitari de Terrassa (CST), Av Pau Casals, 12, 08755 Castellbisbal, Spain; 2 Primary Health Care, CAP St. Genís (CST), Carrer Miquel Mumany, 11, 19, 08191 Rubí, Spain; mcgarciaa@cst.cat (M.C.G.-A.);; 3 Primary Health Care, CAP St. Llàtzer–Centre Universitari (CST), c/ de la Riba 62, 08221 Terrassa, Spain; mgragea@cst.cat; 4 Primary Health Care, CAP Can Roca (CST), c/ Fàtima 18, 08225 Terrassa, Spain; mllistosella@cst.cat; 5 Primary Health Care, CAP Anton de Borja-Centre Universitari, Consorci Sanitari de Terrassa (CST), c/Marconi-Cantonada Edison s/n, 08191 Rubí, Spain; 6 Human Anatomy and Embryology Unit, Faculty of Medicine, Universitat de Barcelona, c/Casanova 143, 08036 Barcelona, Spain; 7 Research and Innovation, Hospital de Terrasa-Hospital Universitari (CST), Carretera de Torrebonica s/n, 08227 Terrassa, Spain

**Keywords:** long COVID, thrombosis, vaccine, COVID-19, prevalence

## Abstract

Background: Long COVID can persist for years, but little is known about its prevalence in relation to the number of infections. This study examines the prevalence of long COVID in association with the number of infections and vaccination status. Methods: We analyzed anonymized data on long COVID cases, thrombotic events and polypharmacy from March 2020, provided by the Data Analysis Control Department for the population assigned to the CST (192,651 at March 2025). Additionally, we analyzed responses to a long COVID symptom-specific survey distributed in March 2024 to individuals aged 18 to 75 years from the CST population diagnosed with COVID-19 as of December 2023 (*n* = 43,398; 3227 respondents). Symptomatic patients suspected of having long COVID underwent blood tests to exclude alternative diagnoses. Results: The overall detected prevalence of long COVID was 2.4‰, with higher frequency among women aged 30–59 years (*p* < 0.001). The survey, combined with specific blood tests, improved detection rates by 26.3%. Long COVID prevalence was 3–10 times higher in individuals with three or more infections than in those with only one recorded infection (based on survey/CST data, respectively). The absolute number of thrombotic events among individuals aged >60 doubled from 2020 to 2024, occurring in both vaccinated and unvaccinated individuals, as well as in those with or without prior documented COVID-19 infection, including in patients without chronic treatments. Conclusions: We found a link between SARS-CoV-2 reinfection and long COVID, and a post-pandemic rise in thrombotic events across all populations, regardless of vaccination or prior infection. Findings support continued COVID-19 diagnosis in suspected cases and mask use by healthcare workers treating respiratory patients.

## 1. Introduction

Long COVID has been defined as ‘the continuation or development of new symptoms 3 months after the initial SARS-CoV-2 infection, with these symptoms lasting for at least 2 months with no other explanation’ [1]. The appearance of a wide variety of persistent symptoms after a COVID-19 infection was described early during the pandemic and included neuropsychiatric, cardiovascular, gastrointestinal, hepatobiliary, and renal sequelae, as well as multisystem inflammatory syndrome in children [2,3], and has been extensively reviewed [4].

Cognitive impairment, including brain fog, may manifest as difficulties with concentration, memory, receptive language, and/or executive function, even after a mild infection [5,6,7]. Dyspnea, pain symptoms, headache, arthralgia, and myalgia, as well as loss of taste and smell, may also persist for months [8].

Long COVID symptoms [9] and cardiovascular sequelae could also be present even after mild or asymptomatic SARS-CoV-2 infection in young people: up to 15% of myocarditis and 30.9% of myocardial injury cases were found after performing MRI in competitive college athletes [10]. Some authors have considered that long COVID also encompasses multiple adverse outcomes, with common new-onset conditions including cardiovascular, thrombotic and cerebrovascular disease [3]. The involvement of the cardiovascular system by SARS-CoV-2 infection includes cardiac arrest, heart failure, myocardial inflammation, stroke, endothelial dysfunction, microangiopathy, and hematological conditions such as coagulopathy, deep vein thrombosis, microclots, and endothelial irregularities [11]. Several biological mechanisms are implicated in hyperinflammation and thrombosis, key factors in COVID-19 severity and long COVID [12]. On the other hand, the newly developed mRNA COVID-19 vaccines [13,14,15,16] also showed increased cardiovascular risk [17,18,19,20], but the benefits of vaccination in preventing severe COVID-19 outcomes have been considered to outweigh the potential complications [19]. Either COVID-19 or vaccination have also been involved in autoimmune diseases [21,22,23].

Several studies have reviewed the prevalence of long COVID, reporting that 10–30% of hospitalized COVID-19 survivors might develop long COVID [24], and most conclude that symptoms may persist for over a year [25,26]. However, there is a gap in knowledge regarding the long-term persistence of symptoms, as well as their relationship with multiple infections and vaccination, which needs to be addressed. Recent studies suggest an increase in long COVID prevalence depending on the number of infections [27,28].

The present study assesses the prevalence of long COVID in an integrated public healthcare consortium—which comprises eight primary healthcare centers, one long-term care center and a referral hospital—analyzing the number of infections, vaccination status, and persistent or transient symptoms affecting functional status.

## 2. Materials and Methods

The cross-sectional study of long COVID-19 prevalence in the Consorci Sanitari de Terrassa (CST) (Ref 02-24-156-028) was approved by the Ethics Committee of the CST on 29 January 2024, while the observational clinical trial registered on 29 April 2020 (NCT04367883) (https://clinicaltrials.gov/study/NCT05504057, accessed on 30 June 2025) was expanded to include long COVID syndrome and thrombotic events on 24 February 2025. The planning, conduct, and reporting of the study adhered to the principles of the Declaration of Helsinki.

All assisted patients signed informed consent indicating whether they accept or decline receiving SMS messages from the CST on their contact phone.

### 2.1. Socioeconomic Environment

The details of the socioeconomic characteristics of the CST have been published previously. Briefly, the CST is a public healthcare organization serving 192,651 residents (as of March 2025) in the North Metropolitan Barcelona Health Region. Its network of centers operates in rural, residential, and metropolitan settings. Despite socioeconomic differences across areas, previous reports have shown consistently high pre-pandemic life expectancy (over 81 years), COVID-19 vaccination rates above 90% among older adults with multiple chronic conditions, and similar infection rates (22–27%). The population aged over 60 ranges from 15.1% to 24%, with most centers above 20% [29,30].

### 2.2. Quantification of Long COVID Syndrome Prevalence and Thrombosis

The Data Analysis Control Department collected anonymized data on COVID-19 cases, hospitalizations, long COVID syndrome, and thrombotic events from March 2020 to March 2025, along with data on gender, age, number of chronic treatments, and COVID-19 vaccination status prior to the first infection in the CST population. Data on cases and hospitalizations have been analyzed previously [29,30,31]. Here, we will focus on long COVID syndrome and thrombosis incidence as part as long-term effects of a COVID-19 infection. Details on strokes, myocardial infarction, pulmonary thromboembolism, and retinal vessel thrombosis will appear in a separate article. All are grouped here as thrombotic events. The entire study population was included in the analysis without exclusions.

### 2.3. Survey: Persistent Symptoms

A link to an online survey was sent to individuals aged 18 to 75 years from the CST population diagnosed with COVID-19 as of December 2023 (*n* = 43,398) via SMS on 19 April 2024. Individuals above and below these ages and those who did not consent to receive SMS were excluded. The questionnaire is available in Supplementary Materials S1. Medical records of patients who gave their consent to be contacted (1546) were reviewed. If no preexisting morbidity explaining the symptoms was identified, an analysis was performed to exclude incident pathology (including general blood test and specific profiles depending on the symptoms reported) (Supplementary Materials S2), as suggested in international guidelines [32,33] (updated January 2024). If the analysis did not lead to new diagnoses, a long COVID diagnosis was added to the medical record.

The part of the team responsible for survey preparation consisted of two physicians and one nurse specialized in primary care and included questions about the symptoms recommended by the Catalan Primary Care Society [32], based on a NICE protocol [33], with acknowledgment of the Ph.D. members of the team. No pilot survey was sent, but a first SMS warned the population that a second SMS, a few days later, would include the survey. The survey was available online for one month. Anonymized survey data were analyzed by one of the researchers, independently and blinded to the part of the team that contacted patients.

### 2.4. Statistical Analysis

Numbers of COVID-19-infected patients were analyzed, excluding duplicate codification of the same episode by different healthcare workers by excluding infections whose infection date differed in less than 45 days of another infection.

Patients were stratified by gender, age (≤60 or >60 years), number of SARS-CoV-2 infections, and vaccination status prior to the first SARS-CoV-2 infection. The prevalence of long COVID across subgroups—defined by age, number of infections, and vaccination status—was compared using OpenEpi chi-square tests when specific categories showed at least 5 cases [34]. Additionally, paired Student’s *t*-tests were employed to analyze changes in self-reported health perception before and after SARS-CoV-2 infection.

## 3. Results

### 3.1. Prevalence of Long COVID and Number of Infections

The overall detected prevalence of long COVID is 2.4‰ (3.3‰ in women vs. 1.6‰ in men, *p* < 0.0000001). The survey has allowed for an increase of 26.3% in the detection of affected patients, identifying 99 new cases compared to the previous 376, resulting in 475 among the population of 192,651 residents assigned to the CST at March 2025 (Table 1).

**Figure 1:**
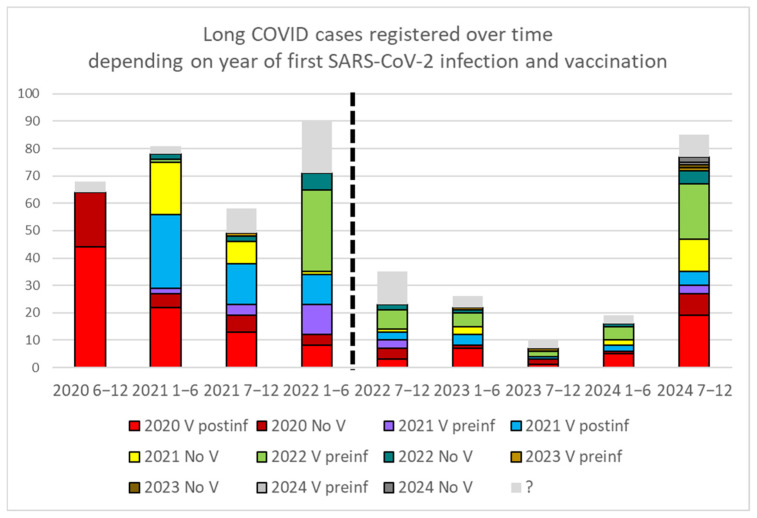
Long COVID cases detected every six months (1–6 indicate the first half of the year, and 7–12 the second half). The year of the first COVID-19 infection is also shown, indicating whether vaccination occurred prior to infection (V preinf), after infection (V postinf), or not at all (No V). The dashed line marks the end of the protocol recommending testing for any symptomatic patient. The increase observed in 2024 coincides with active case finding in the CST via survey. In some long COVID records, the exact date of infection is uncertain, likely due to previous unreported self-diagnosis by the patient. Those cases with uncertain data of infection are indicated as ‘?’.

**Figure 2:**
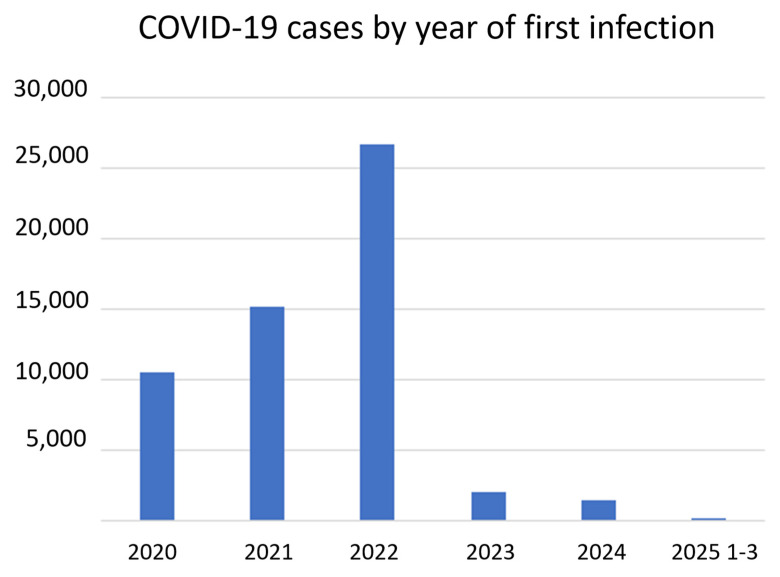
Period of the first SARS-CoV-2 infection. The number of detected cases decreased dramatically in March 2022, after the end of protocols that required case detection beyond symptomatic patients.

Most of the long COVID cases corresponded to infections that occurred in 2020 (Figure 1). The number of registered cases decreased progressively until the survey was launched in 2024. The decline in long COVID diagnoses paralleled the decrease in the number of COVID-19 cases following the end of protocols that required test confirmation for any symptomatic patient and their close contacts in March 2022 (Figure 2). The launch of the survey in 2024 led to the detection of additional symptomatic patients after suffering COVID-19, who consented to be contacted and had their medical records reviewed. These patients had no preexisting pathologies explaining their symptoms, and newly scheduled blood tests excluded other pathologies.

Based on CST registry data, patients with multiple infections were recorded as follows: 49,914 (one infection), 5190 (two), 480 (three), 61 (four), 14 (five), 3 (six), 2 (seven), and 1 (eight multiple infections).

Long COVID prevalence increased significantly with the number of infections, showing an odds ratio (OR) of >10 across all groups suffering three or more infections compared to a single infection. It was also higher among those who received the first vaccine dose after their first COVID-19 infection (Table 2). LC prevalence related to number of doses of vaccine received is presented in Table S3.

### 3.2. Survey: Reported Persistent and Transient Symptoms

In total, 21.7% (702) of 3227 survey respondents reported being diagnosed with long COVID, while 16.3% were unsure about this diagnosis. Reported symptoms are summarized in Table 3 and detailed per language in Table S2A. Among those diagnosed with long COVID, 64% reported being currently symptomatic. All respondent groups rated their overall health perception above 8 points out of 10 prior to COVID-19 infection (8.4 ± 1.5 on average). Those who felt recovered rated their current health at 6.9 ± 1.8 vs. 8.2 ± 1.4 pre-infection (*p* < 0.001), while those with persistent symptoms rated theirs at 5.04 ± 2.02 vs. 8.1 ± 1.95 pre-infection (*p* < 0.001).

The most frequently reported symptoms of long COVID (≥33%) were persistent fatigue, joint pain, and lack of concentration. However, more than 20% of patients who had experienced these symptoms reported feeling well by the time the survey was launched. Detailed responses stratified by gender are provided in the Table S1, with no major differences observed between genders.

A total of 1497 respondents (46.4%) needed COVID-19-related sick leave. Most were on leave for <1 month (77.2%), 12.0% for 1–3 months, 2.7% for up to 6 months, 2.6% for up to 1 year, and 5.3% for more than 1 year.

The percentage of long COVID relative to the number of SARS-CoV-2 infections and vaccines received among survey respondents is detailed in Table 4 and Table S2B. Long COVID prevalence increased with infection number in both unvaccinated (from 9.1% to 30.7%) and vaccinated (from 10.6% to 25.4%, *p* < 0.0001) respondents when comparing 1 infection with ≥3 infections.

### 3.3. Thrombotic Events

**Figure 3:**
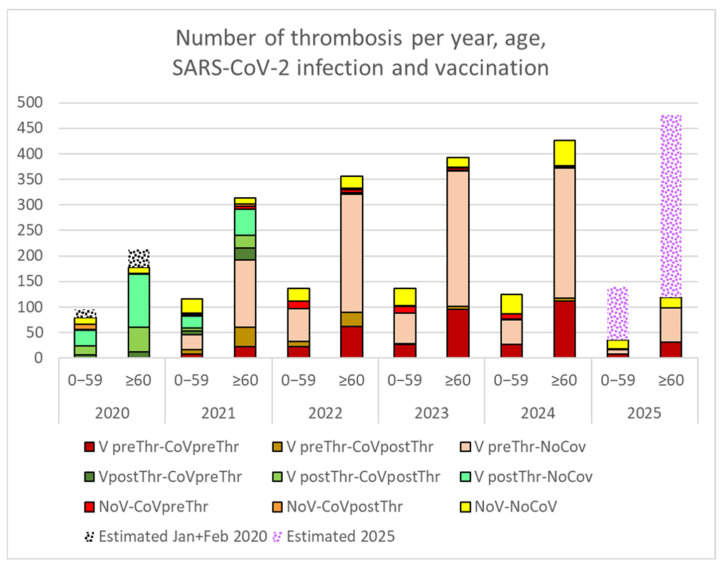
Cumulative thrombotic events from March 2020 to March 2025, stratified by age (≥60 vs. <60 years), vaccination status (vaccinated [V] vs. non-vaccinated [NoV]) and SARS-CoV-2 infection (CoV or NoCoV, together with the temporal relation to thrombotic events, either before (preThr) or after (postThr) the event). Annual thrombosis estimates (2020, 2025) were extrapolated from monthly averages of recorded events, adjusting for months with missing data.

Anonymized data from CST patients with thrombosis from March 2020 show a linear increase in this phenomenon (Figure 3), particularly in patients aged over 60 years.

Increased incidence is evident among those with prior COVID-19 infection and unvaccinated individuals without detected infection.

Appendix A presents detailed figures on the interplay between thrombosis, COVID-19 infection, vaccination status, and polypharmacy.

The increase in thrombosis is significant in both vaccinated individuals and those without vaccination records (Table 5).

An increase in thrombotic events was also evident among patients with prior COVID-19 infection, as well as those without any documented infection (Table 6).

## 4. Discussion

This study confirms previous reports of an increased prevalence of long COVID after repeated SARS-CoV-2 infections [27,28] and describes a substantial rise in thrombotic events following the pandemic.

### 4.1. Increased Prevalence with Multiple Infections

To our knowledge, this is the study with the longest follow-up confirming a significant association between the number of SARS-CoV-2 infections and increased long COVID prevalence. Both anonymized population-wide data and survey-based analyses consistently revealed higher prevalence rates in individuals with three or more recorded infections compared to those with only one infection.

Data extracted from electronic medical records by the Management, Control, and Information Analysis Unit—coded by primary care physicians—revealed a tenfold increase in long COVID prevalence among individuals with three or more infections. Prevalence exceeded 2% in those vaccinated prior to their first infection and was even higher in other groups (e.g., unvaccinated: OR 1.30).

This trend was corroborated by survey respondents, where individuals reporting three or more infections exhibited triple the rate of persistent symptoms (affecting > 20% across all groups) compared to those with only one infection, regardless of vaccination status. While potential respondent bias may influence survey results [9,27,35], the consistency with the CTS’s recorded cases strengthens the evidence for a dose-dependent relationship between reinfection and long COVID risk.

The 702 survey respondents reporting a long COVID diagnosis exceed the 376 patients recorded with this diagnosis in the CST registry prior to the survey launch (see Table 1 and Table 4). This suggests not only a high response rate among symptomatic patients but also indicates potential respondent bias, as reported in previous long COVID studies [35]. However, the amount of missing data appears minimal [36], supporting the robustness of the prevalence ratios. A review of symptomatic patients’ histories and specific blood tests, used to rule out other pathologies, led to the recoding of 99 additional long COVID cases.

However, the overall low response rate (3227 respondents out of nearly 44,000 diagnosed patients) contrasts with the high participation among long-term affected individuals. This discrepancy may result in an overestimation of secondary outcomes—such as percentage of symptom persistence and sick leave rates—also due to respondent bias [35].

Since multiple immunological mechanisms have been described to be impaired after SARS-CoV-2 infection and may contribute to the development of long COVID [12], it is likely that this impairment is exacerbated by repeated infections.

### 4.2. Increase in Thrombotic Events

To our knowledge, this is also the first study to demonstrate a linear increase in thrombotic events over the past five years, with rates doubling since 2020 among patients aged ≥60 years. This rise was most pronounced in individuals with documented COVID-19 infections but remained significant even in those without recorded infections or vaccinations, potentially reflecting undiagnosed mild or asymptomatic cases following the discontinuation of universal testing protocols. Furthermore, vaccination data may be incomplete for younger individuals, as multiple vaccination sites operated outside the reference institution. Recorded vaccination rates exceed 90% in patients aged >60 years on ≥2 chronic treatments but are likely underestimated in younger, less polypharmacy-exposed groups. Detailed analyses of COVID-19 reinfections, multiple vaccinations, polypharmacy patterns, and thrombosis subtypes will be presented in a separate article.

While our findings should be interpreted cautiously due to a 15% increase in the assigned population (resulting from a healthcare restructuring, including the addition of a new primary care center in Terrassa in October 2022), this demographic shift cannot account for the initial rise in thrombotic events observed as early as 2021, nor the magnitude of the increase (approximately 100% by 2024 compared to 2020 in those aged >60).

Although global hospital admissions for acute coronary syndrome (ACS) declined during the early pandemic phase—attributed to patient reluctance to seek care and undiagnosed cases—studies anticipated both short- and long-term complications of myocardial infarction [37]. Part of the anticipated increase in coronary syndromes could be attributed to reduced preventive care during the early stages of the pandemic due to restrictions. However, since most primary care activity had been restored by 2022, this factor cannot explain the continued linear increase in subsequent years. Although detailed comorbidity data are not available, patients aged >60 years without any treated chronic conditions showed a significant increase (2.3 fold), as did those aged 40–60 years (+20%), though the limited sample size precluded statistical significance. Surprisingly, the most comorbid population (patients receiving ≥8 chronic treatments) showed no increase. If certain immune responses to COVID-19 infection (see below) or vaccination were related to the thrombotic increase, the impaired immunity often associated with highly comorbid patients might explain this finding.

Multiple biological mechanisms associated with COVID-19 infection may explain the increased thrombotic risk, driving endothelial dysfunction and multiorgan damage [12], while recurrent infections are linked to disrupted homocysteine metabolism, perpetuating cycles of inflammation and hypercoagulability [38].

### 4.3. Persistent and Transient Symptoms—Functional Implications

The temporal alignment between the date of first COVID-19 infection and long COVID diagnosis aligns with studies documenting its persistence beyond one year [9,25,26,27,39] (among others), with the present results suggesting symptoms may last for over four years. Our finding of higher long COVID frequency in females corroborates previous reports [25,26,37,38,39].

Given the wide range of persistent symptoms reported after long COVID, we focused on specific manifestations (anosmia/dysgeusia) and general symptoms (joint pain, fatigue, memory loss, poor concentration) emerging post-infection. These align with prior studies [26] and meta-analyses [4]. We actively evaluated patients who reported post-COVID-19 symptoms but were uncertain about a long COVID diagnosis. Those consenting to contact underwent targeted blood tests to exclude alternative pathologies; cases without confirmed alternatives were coded as long COVID. To our knowledge, this is the largest study actively screening undiagnosed long COVID patients in a ~55,000-resident population, increasing detection by 26%.

While many patients experienced persistent symptoms, some reported gradual recovery; however, overall self-rated health remained significantly impaired compared to pre-infection levels. This pattern mirrors studies describing partial improvement over time despite lasting health impacts [39,40].

Our finding that nearly half of respondents required sick leave aligns with prior studies identifying work incapacity as a common functional consequence of long COVID [25]. Although respondent bias, evidenced by higher participation rates among severely affected patients, could impact prevalence estimates, the comparable absolute numbers (survey-reported vs. registry-coded long COVID cases) and consistency with established literature suggest this figure remains plausible. Finally, fewer than 5% needed extended sick leave (>1 year), consistent with evidence of gradual functional improvement over time and with the original definition of ‘post-COVID-19 syndrome’ in 2021 as a ‘concurrence of a multisystem, fluctuating, and often overlapping clusters of signs and symptoms that, in some patients, may follow a relapsing-remitting pattern and that may change over time [32]’.

### 4.4. Long COVID, Public Health and Future

Given that 20% of long COVID patients require sick leave exceeding one month—coupled with rising prevalence post-reinfection—it remains unclear whether persistent functional impairment will increase with future infections or if spontaneous recovery will stabilize population functionality.

From the pandemic’s early stages, multiple drugs have been explored for repurposing [41], including antihistamines and antiparkinsonian agents. Primary care reports describe empirical antihistamine use to prevent post-COVID-19 syndrome [42], while amantadine—an antiparkinsonian drug with historical use as an influenza antiviral—has demonstrated efficacy in alleviating fatigue [43] and depressive symptoms [44]. Notably, metformin, a commonly prescribed medication, exhibits broad-spectrum antiviral activity against RNA viruses, reducing hospitalization/mortality rates [45] and lowering long COVID incidence by approximately 40% [46].

Randomized controlled trials have evaluated diverse interventions for post-COVID-19 syndrome: biologic drugs designed to clear extracellular RNA from latent reservoirs show promise in reducing fatigue by improving chronic inflammation [47]; antiviral agents yield mixed results, with ensitrelvir demonstrating efficacy [48] but nirmatrelvir-ritonavir showing no benefit [49]; and micronutrient supplementation likewise failed to improve outcomes [50].

Additional therapeutic approaches include rehabilitation, whether supervised telerehabilitation [51], asynchronous [52], or face-to-face [53], all of which have demonstrated clinical benefits, as well as cognitive exercises [54]. In contrast, cognitive interventions remain limited in scope, though future strategies may incorporate combined neurostimulation and cognitive training [55].

Further investigation is ongoing since COVID-19 hospitalizations continue (Figure S1), with variant XFG in 58% of random samples of symptomatic patients [56], with incidence in June 2025 being similar to that of January 2025.

The increase in long COVID cases following multiple reinfections suggests that public health recommendations aimed at reducing viral transmission should remain in place—such as the use of protective masks by symptomatic individuals and the healthcare workers attending to them. Moreover, the rising incidence of thrombosis is expected to pose a significant public health threat, with substantial economic consequences related to treatment costs and reduced quality of life. Specific tests to detect SARS-CoV-2 antibodies resulting from infection (anti-nucleocapsid), as opposed to vaccination [57], would be useful in reassessing a patient’s individual risk. These antibodies could also support the diagnosis of long COVID in symptomatic individuals who may have experienced undocumented infections after 2022.

### 4.5. Limitations of the Study

As previously reported [31], the limitations of the study include the following: (1) the inability to precisely quantify first-wave cases due to diagnostic test shortages in primary care until June 2020; (2) the overall 78% sensitivity of publicly available antigen tests (introduced November 2020) [58]; and (3) the discontinuation of systematic COVID-19 testing protocols for symptomatic patients after 24 March 2022 [59], which precipitated a sharp decline in detected cases despite sustained weekly hospital admissions through January 2025 [29,30]. Consequently, both COVID-19 infection rates and syndrome incidence could be underestimated, with undocumented infections potentially contributing to undiagnosed cases. Nevertheless, such bias could affect equally the comparison of the prevalence of long COVID between the groups regarding the number of registered infections.

Another factor of confusion for the calculation of long COVID prevalence is that both long COVID (‘COVID persistent’) and COVID-19 sequelae share the same ICD-10-CM code (U09.9) in the medical software for history records. Furthermore, although the medical record software allows clinicians to close open diagnoses once symptoms are resolved, a long COVID diagnosis will likely remain open until actively closed during a follow-up consultation with the patient’s primary care physician. This follow-up is unlikely to occur if the patient feels better. Furthermore, clinicians might omit this step to save time during the visit. Consequently, the current absolute prevalence could overestimate the symptomatic population. Other groups [60,61] have also reported those codification difficulties.

An additional limitation involves decentralized vaccination in 2021 for individuals under 60 years, which may have led to incomplete records in CST vaccination registries—despite >90% vaccination coverage in those over 60 at primary care centers during early 2021. On the other hand, the contact phone number of older patients coincided with the phone number of younger relatives, leading to a potential underestimation of respondents in older ages.

Data comparability with other teams from surrounding institutions confirm the decline in symptomatology after several months [39], although regional results may be influenced by Spain’s unique variant evolution early in the pandemic, particularly the dominance of the B3a strain, which was uncommon elsewhere in Europe [62]. Further research should investigate whether this distinct variant’s virulence contributes to divergent long COVID manifestations.

## 5. Conclusions

Our findings demonstrate a relationship between SARS-CoV-2 reinfection and long COVID prevalence. Additionally, we observed a significant post-pandemic rise in thrombotic events across all populations, regardless of vaccination status or documented prior COVID-19 infection. Results suggest that COVID-19 diagnosis and spread prevention should continue in suspected cases to reassess future long COVID cases and cardiovascular risk, and that healthcare workers attending respiratory patients should continue wearing protective masks.

## Figures and Tables

**Table 1:** Prevalence of long COVID by age and gender among the CST population.

	Long COVID		Total		
	Pre Survey	Post Survey	Long COVID	No Long COVID	Population
**Women/age**	**242**	**+80**	**322 (3.3‰)**	**96,676**	**96,998**
0–29	19	+2	21 (0.7‰)	30,907	30,928
30–59	171	+63	234 (5.6‰)	41,530	41,764
≥60	52	15	67 (2.7‰)	24,239	24,306
**Men/age**	**134**	**+19**	**153 (1.6‰)**	**95,500**	**95,653**
0–29	16	+1	17 (0.5‰)	32,850	32,867
30–59	90	+11	101 (2.3‰)	43,054	43,155
≥60	28	+7	35 (1.7‰)	19,596	19,631
**Total general**	376	+99	**475 (2.4‰)**	**192,176**	**192,651**

The additional number of new cases detected after the survey is detailed in the ‘post survey’ column.

**Table 2:** COVID (CoV) and Long COVID registered cases (LC) depending on the number of registered infections (*n* infections) and vaccination (V).

*n* Infection	V	CoV	LC	(%)	OR vs. V preinf	*p*	OR vs. 1 inf	*p*
?	V	60,985	45	0.07%				
?	No V	75,664	21	0.03%				
1	V preinf	18,386	46	0.25%				
1	V postinf	9889	168	1.67%	6.68	<0.0001 *		
1	No V	21,608	94	0.43%	1.8	0.001 *		
2	V preinf	2948	43	1.44%			5.8	<0.0001 *
2	V postinf	335	14	4.01%	2.78	0.004 *	2.4	0.001 *
2	No V	1833	18	0.97%	0.67	0.07	2.2	0.001 *
≥3	V preinf	355	10	2.74%			11	<0.0001 *
≥3	V postinf	37	8	17.78%	6.49	<0.0001 *	10.6	<0.0001 *
≥3	No V	136	8	5.56%	2.03	0.06	12.8	<0.0001 *

Cases without a confirmed infection date are marked as (?) and vaccination is separated in those having received at least one vaccine prior (V preinf) or after (V postinf) the first COVID-19 infection. Significant *p* are indicated with *.

**Table 3:** Percentage of each symptom among patients reporting long COVID diagnoses and percentage of patients referring spontaneous recovery.

Responders with Long COVID Diagnosis:	Yes 702	Yes, and I Am Still Symptomatic	Yes, but I Already Feel Good		Unsure 527	No 1998
Physical complaints						
Anosmia or dysgeusia	23.5%	165	35	17.5%	76	76
Shortness of breath	31.9%	224	47	17.3%	158	136
Headache	25.1%	176	48	21.4%	155	170
Joint pain	36.5%	256	103	28.7%	259	332
Persistent fatigue	46.9%	329	123	27.2%	323	406
Psychological complaints						
Memory complaints	31.9%	224	65	22.5%	164	198
Lack of concentration	33.3%	234	61	20.7%	190	224
Depression	19.2%	135	38	22.0%	119	144
Anxiety	33.2%	233	87	27.2%	219	303
Sleep complaints	32.3%	227	80	26.1%	215	299
Functional impairment						
Home task	23.1%	162	31	16.1%	107	109
With friends or relatives	17.9%	126	24	16.0%	90	132
Impaired personal hygiene	5.4%	38	9	19.1%	26	33
Work interference	29.3%	206	53	20.5%	144	165
COVID-19-related sick leave	37.7%	265	132	33.2%	244	1002

Absolute numbers of respondents uncertain about their diagnosis and respondents without long COVID are also included.

**Table 4:** Percentages of responders reporting long COVID syndrome are related to the number of reported COVID-19 infections and the number of vaccines received.

Number of Infections	Vaccination				Number of Vaccines							
	No Vaccines		At Least 1		1		2		3		>3	
1 COVID-19 infection	77		1753		143		691		615		304	
No	57		1176		90		439		434		213	
Unsure	8		277		17		125		95		40	
Long COVID still symptomatic	7	9.1%	185	10.6%	21	14.6%	81	11.7%	51	8.2%	33	10.8%
Transient long COVID	5	6.5%	114	6.5%	15	10.5%	46	6.7%	35	5.7%	18	5.9%
2 COVID-19 infections	47		962		98		401		356		107	
No	27		540		47		212		216		65	
Unsure	6		174		13		76		64		21	
Long COVID still symptomatic	9	19.14%	167	17.3%	30	30.6%	81	20.1%	45	12.6%	11	10.2%
Transient long COVID	5	10.6%	81	8.4%	8	8.2%	32	10.5%	31	8.7%	10	9.3%
≥3 COVID 19 infections	13		173		55		118		99		34	
No	6		78		21		56		42		15	
Unsure	0		30		12		20		16		6	
Long COVID still symptomatic	4	30.7%	43	25.4%	18	32.7%	29	24.5%	23	23.2%	8	23.5%
Transient long COVID	3	23.1%	22	12.7%	4	7.3%	13	11.0%	18	18.2%	5	14.7%

**Table 5:** Number of thrombosis cases in vaccinated (V) and unvaccinated (No V) patients. Some patients received the vaccine after the thrombotic event (V post Thr), and others before the event (V pre Thr). Odds ratios (ORs) compare thrombosis incidence in 2024 versus 2021—the first and last years with complete data—with significant differences observed in both vaccinated and unvaccinated groups.

	2020	2021	2022	2023	2024	2025	No Thrombus	OR	*p*
V								2024-21	
V post Thr	219	137	3	1	3	1			
V pre Thr		237	418	454	447	114		1.89	0.0000001 *
							91,235		
No V	37	55	72	74	100	39	99,005	1.82	0.0003 *

Some patients received the vaccine after the thrombotic event (V post Thr), and others before the event (V pre Thr). Odds ratios (ORs) compare thrombosis incidence in 2024 versus 2021—the first and last years with complete data—with significant differences (*) observed in both vaccinated and un-vaccinated groups.

**Table 6:** Number of thrombotic events in Vaccinated (V), non vaccinated (No V) and SARS-CoV-2 infection

	Thrombus						No Thrombus	OR 2024-21	*p*
	**2020**	**2021**	**2022**	**2023**	**2024**	**2025**			
V									
CoV									
CoV post Thr	67	79	37	7	5				
CoV pre Thr	18	62	86	123	139	39		2.24	0.0000001 *
No thrombus							31,577		
No Cov	134	233	298	325	306	76	59,658	1.31	0.0017 *
No V									
CoV	13	16	24	21	14	2	23,607		
CoV post Thr	11	7	4	3					
CoV pre Thr	2	9	20	18	14	2		1.55	0.29
No thrombus							23,607		
No Cov	24	39	48	53	86	37	75,398	2.20	0.0002 *
Total general	256	429	493	529	550	154	190,240		

Some patients experienced COVID-19 infection prior to thrombosis (CoV pre Thr), while others suffered it after the thrombotic event (CoV post Thr). A third group had no reported COVID-19 infections (No CoV). Significant *p* are indicated with *.

**Figure A1:**
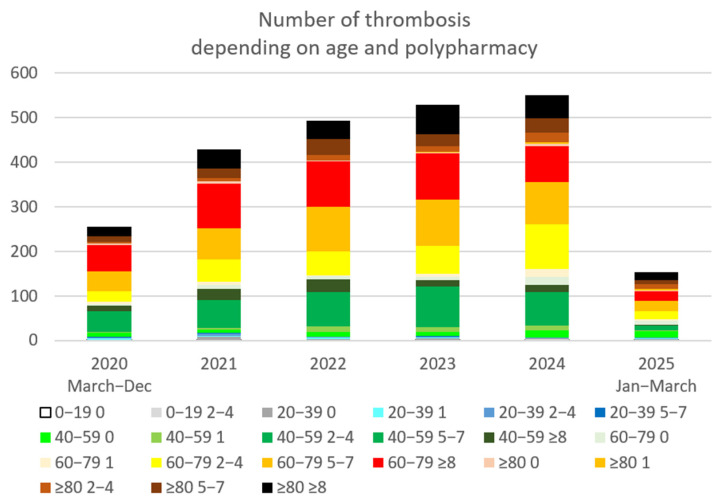
Annual thrombosis incidence (March 2020–March 2025), stratified by 20-year age groups and number of chronic treatments (0, 1, 2–4, 5–7, ≥8).

**Figure A2:**
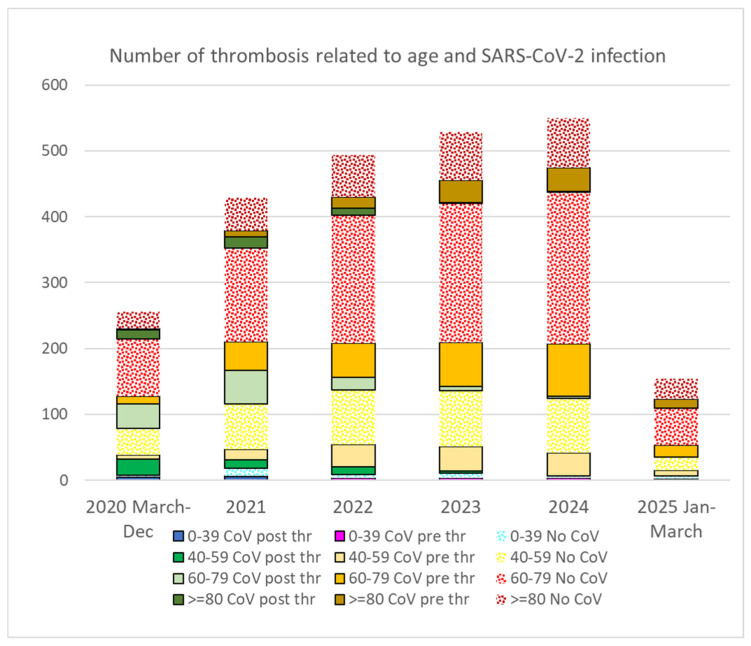
Thrombosis events linked to COVID-19 infection timeline. Warm colors indicate the first infection preceding thrombosis (CoV pre thr) while cool colors indicate infection after the thrombosis (CoV post thr). Dashed border indicates thrombosis without documented infection. (Follows pre-thr color coding).

**Figure A3:**
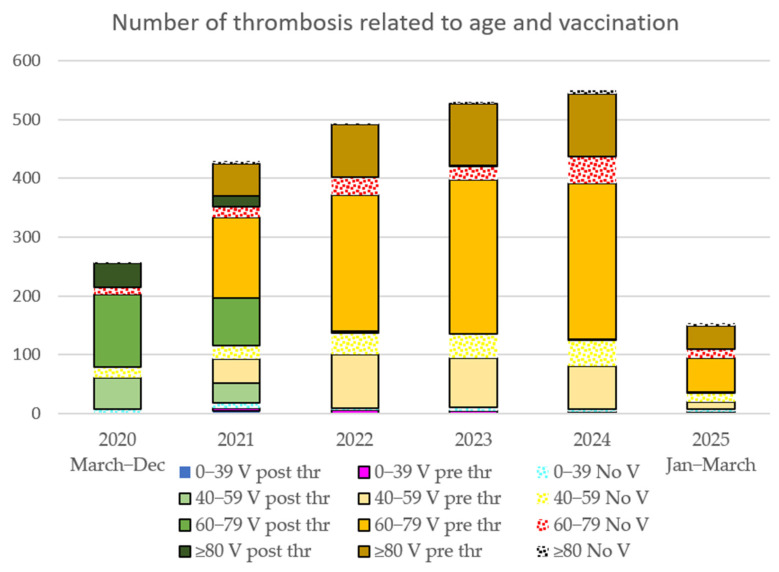
Thrombosis events linked to vaccination. Warm colors indicate at least one vaccination preceding thrombosis (V pre thr) while cool colors indicate vaccination after the thrombosis (V post thr). Dashed border indicate thrombosis without documented vaccination. (Follows pre-thr color coding).

## Data Availability

The original contributions presented in this study are included in the article. Further inquiries can be directed to the corresponding author.

## Supplementary Materials

The following supporting information can be downloaded at: https://www.mdpi.com/article/10.3390/vaccines13090905/s1. The survey questionnaire, the specific blood test according to symptoms, the answers per gender and language, the % of Long COVID related to the numbers of vaccines according to the CST registers and number of COVID-19 hospitalizations in the first semester of 2025 are presented in Supplementary Materials.

## Abbreviations

The following abbreviations are used in this manuscript:

LC	Long COVID
No V	Non-vaccinated
Preinf	Previous to the COVID-19 infection
Postinf	Posterior to the COVID-19 infection
Pre Thr	Previous to the thrombosis
Post Thr	Posterior to the thrombosis
CoV	COVID-19 infection
CST	Consorci Sanitari de Terrassa

## Appendix A

**Table A1:** Thrombotic events stratified by 20-year age groups and number of chronic treatments. Odds ratios (ORs) for thrombosis occurrence (2024 vs. 2021) are shown, together with *p*-values (reported as * when significant).

Age-Polypharmacy		2020	2021	2022	2023	2024	2025	No Thrombus	OR 2024-21/*p*
	Year of Thrombosis								
0–39									
0		2	5	2	4	3	3	76,579	0.6
1		2	1	1	3		1	7205	0.0
2–4		1	2	3	1	3	2	3723	1.5
5–7		2	1		2	1	1	281	1.0
≥8								53	
40–59									
0		7	11	10	7	13	13	35,889	1.2
1			3	13	9	10	3	9748	3.3 * (*p* = 0.02)
2–4		16	29	40	42	43	10	11,572	1.5 * (*p* = 0.026)
5–7		17	21	21	44	28	1	2549	1.3
≥8		7	15	21	9	15	1	796	1.0
60–79									
0		6	9	9	7	21	9	6642	2.3 * (*p* = 0.04)
1		5	5	3	8	19	3	4270	3.8 * (*p* = 0.004)
2–4		26	54	55	64	101	18	12,044	1.9 * (*p* = 0.002)
5–7		46	68	93	94	89	24	6653	1.3 * (*p* = 0.047)
≥8		49	92	90	95	74	21	3715	0.8
≥80									
0		3	6	2	3	4	3	509	0.7
1		1	1		2	5	2	338	5.0
2–4		8	12	19	15	22	11	2147	1.8 * (*p* = 0.04)
5–7		19	31	51	39	40	9	2785	1.3
≥8		39	63	60	81	59	19	2742	0.9
Total general		256	429	493	529	550	154	190,240	1.3 * (*p* = 0.0001)
