# Supplementary material for: Long COVID Syndrome Prevalence in 2025 in an Integral Healthcare Consortium in the Metropolitan Area of Barcelona: Persistent and Transient Symptoms
Source: Vaccines (Basel). 2025 Aug 26;13(9):905. doi: 10.3390/vaccines13090905 (PMC12474489; doi:10.3390/vaccines13090905)

Supplementary Material S1

English translation of the survey questionnaire, that was sent in either catalan and Spanish: Since 2020, many people have suffered from COVID-19. The aim of this survey is to assess the current situation from the patients' point of view. We would appreciate it if you could answer this survey, which will take no more than 3 minutes to complete and may help improve your healthcare.

1. Gender
- Woman
- Man
- I identify differently
2. 2. Age
- 18 to 35
- 36 to 50
- 51 to 65
- 66 to 75
- 76 or older
3. 3. Have you received any COVID-19 vaccines?
- No
- Yes. 1 dose
- Yes. 2 doses
- Yes. 3 doses
- Yes. More than 3 doses
4. 4. Have you ever been diagnosed with COVID-19?
- No
- Yes. Once
- Yes. Twice
- Yes. Three or more times

IF YOUR ANSWER IS NO, GO TO QUESTIONS 7 AND 8 AND YOU WILL HAVE COMPLETED THE SURVEY.

5. How was the diagnosis made?
- I had a rapid test
- I had a PCR test
- I had a blood test
- No test was done. Diagnosis based only on symptoms
- I did the test myself

6. Have you been diagnosed with "Long COVID" or had symptoms attributed to COVID that lasted more than 2 or 3 months?
- Yes. I still have symptoms
- Yes, but I am now recovered
- No
- I don’t know

7. Overall rating of your current health status (on a scale from 0 to 10, where 0 is the worst possible health status and 10 is excellent health)
- 0 1 2 3 4 5 6 7 8 9 10

8. Overall rating of the worsening of your health compared to before COVID (on a scale from 0 to 10, where 0 is no change and 10 is maximum worsening)
- 0 1 2 3 4 5 6 7 8 9 10

9. Do you have functional limitations in your daily life (personal hygiene, household tasks, work activity, relationships with friends and/or close family)?
- Yes
- No

10. If you answered yes, please indicate which of the following apply:
- Personal hygiene
- Household tasks
- Work activity
- Relationships with friends and/or close family

11. Are your symptoms mainly physical or psychological?
- Physical symptoms

Alteration in smell or sense of taste
Persistent Fatigue
Joint pain
Headache
Shortness of breath
- Psychological symptoms

Memory loss
Difficulty concentrating
Anxiety
Depression
Sleep compalins
- Both physical and psychological symptoms

12. Have you had to take sick leave for more than a month due to persistent COVID-related symptoms?
- Yes
- No

13. If you answered yes to the previous question, how long were you on sick leave?
- Between 1 and 3 months
- 3 to 6 months
- 6 to 12 months
- More than a year

14. Do you consent to being contacted by your health center (CAP) in case there is any treatment or intervention that could benefit you?
- Yes
- No

15. In that case include the number of your health card.

Supplementay Material S2. General blood test for Long COVID suspected patients:

Complete blood count with white blood cell differential
Glucose
C-reactive protein
Bilirubin
Alanine aminotransferase
Aspartate aminotransferase
Gamma-glutamyl transferase
Alkaline phosphatase
Urea
Sodium
Potassium
Creatine phosphokinase
Lactate dehydrogenase
Albumin
Thyroid-stimulating hormone
Ferritin
Vitamin B12
Folate
Calcium
Phosphorus
D-dimer

Specific profile depending on symptoms:
Myalgia, asthenia, fatigue, articular pain:
Protein electrophoresis
Uric acid
Rheumatoid factor
Antinuclear antibodies
Complement C3
Complement C4
Antiphospholipid antibodies
Cortisol

Dyspnea, thoracic pain:
Pro B-type natriuretic peptide
Amylase

Digestive symptoms:
Amylase
Lipase
Fecal calprotectin
Fecal occult blood
Anti-transglutaminase antibodies

Table S1. Reported symptoms per gender.

Gender	Yes	Yes, and I'm still symptomatic	Yes, but I already feel good	Unsure	No	
Female						
n	515	332	183		342	1229
Anosmia or dysgeusia	22%	112	23	17%	45	45
Shortness of breath	33%	170	34	17%	97	82
Headache	29%	147	35	19%	106	124
Joint pain	37%	189	73	28%	169	225
Persistent Fatigue	49%	250	89	26%	212	267
Memory complains	33%	172	47	21%	110	141
Lack of concentration	35%	181	45	20%	121	146
Depression	20%	102	28	22%	76	90
Anxiety	35%	181	66	27%	154	205
Sleep complains	32%	163	50	23%	141	198
Home task	25%	131	26	17%	74	85
With friends or relatives	19%	99	17	15%	56	82
Impaired personal hygiene	6%	30	6	17%	15	20
Work interference	30%	155	41	21%	84	100
COVID-19 related sick leave	20%	103	57	36%	91	321
Male						
n	175	114	61	35%	174	742
Anosmia or dysgeusia	28%	50	12	19%	30	30
Shortness of breath	28%	50	11	18%	57	53
Headache	15%	27	10	27%	46	42
Joint pain	37%	64	27	30%	85	100
Persistent Fatigue	43%	75	30	29%	104	130
Memory complains	27%	48	16	25%	50	50
Lack of concentration	29%	50	15	23%	64	69
Depression	17%	30	8	21%	43	53
Anxiety	27%	48	18	27%	60	92
Sleep complains	35%	61	17	22%	71	94
Home task	17%	30	2	6%	31	23
With friends or relatives	14%	24	5	17%	32	48
Impaired personal hygiene	4%	7	1	13%	11	13
Work interference	27%	48	10	17%	58	61
COVID-19 related sick leave	19%	33	16	33%	32	207

I identify myself in other gender							
n	12	7	5	42%	11	27	50
Anosmia or dysgeusia	25%	3	0	0%	1	1	5
Shortness of breath	33%	4	2	33%	4	1	11
Headache	16%	2	3	60%	3	4	12
Joint pain	25%	3	3	50%	5	7	18
Persistent Fatigue	33%	4	4	50%	7	9	24
Memory complains	33%	4	2	33%	4	7	17
Lack of concentration	25%	3	1	25%	5	9	18
Depression	25%	3	2	40%	0	1	6
Anxiety	33%	4	3	43%	5	6	18
Sleep complains	25%	3	3	50%	3	7	16
Home task	8%	1	3	75%	2	1	7
With friends or relatives	25%	3	2	40%	2	2	9
Impaired personal hygiene	8%	1	2	67%	0	0	3
Work interference	25%	3	2	40%	2	4	11
COVID-19 related sick leave	16%	2	2	50%	4	13	21

Table S2.A. Responses per language about transient symptoms.

SURVEY IN CATALAN	Yes				Unsure	No
Responders with Long covid diagnosis (CAT)	368	Yes, and I'm still symptomatic (228)	Yes, but I already feel good (140)		281	1222
Physical complains						
Anosmia or dysgeusia	23.4%	86	20	18.9%	40	39
Shortness of breath	27.2%	100	28	21.9%	74	66
Headache	22.3%	82	23	21.9%	75	79
Joint pain	32.9%	121	58	32.4%	135	160
Persistent Fatigue	41.6%	153	68	30.8%	159	209
Psychological complains						
Memory complains	30.7%	113	35	23.6%	91	104
Lack of concentration	31.0%	114	37	24.5%	86	131
Depression	16.6%	61	20	24.7%	52	69
Anxiety	31.3%	115	48	29.4%	101	147
Sleep complains	26.6%	98	34	25.8%	98	151
Functional impairment						
Home task	20.4%	75	19	20.2%	55	52
With friends or relatives	17.4%	64	14	17.9%	42	62
Impaired personal hygiene	4.1%	15	5	25.0%	11	16
Work interference	25.8%	95	35	26.9%	69	79
COVID-19 related sick leave	37.5%	138	75	35.2%	127	541

SURVEY IN SPANISH	Yes			Unsure	No
Responders with Long covid diagnosis (ES):	334	Yes, and I'm still symptomatic (225)	Yes, but I already feel good (109)	246	776
Physical complains					
Anosmia or dysgeusia	23.7%	79	15 (19.0%)	36	37
Shortness of breath	37.1%	124	19 (15.3%)	84	70
Headache	28.1%	94	25 (26.6%)	80	91
Joint pain	40.4%	135	45 (33.3%)	124	172
Persistent Fatigue	52.7%	176	55 (31.3%)	164	197
Psychological complains					
Memory complains	33.2%	111	30 (27.0%)	73	94
Lack of concentration	35.9%	120	24 (20.0%)	104	93
Depression	22.2%	74	18 (24.3%)	67	75
Anxiety	35.3%	118	39 (33.1%)	118	156
Sleep complains	38.6%	129	46 (35.7%)	117	148
Functional impairment					
Home task	26%	87	12 (13.8%)	52	57
With friends or relatives	18.6%	62	10 (16.1%)	48	70
Impaired personal hygiene	6.9%	23	4 (17.4%)	15	17
Work interference	33.2%	111	18 (16.25)	75	86
COVID-19 related sick leave	38%	127	57 (44.9%)	117	461

Table S2.B. Responses per language about respondents with persistent symptoms depending on the number of infections and number of vaccines received.

**ANSWERS IN CATALAN**	**No V**		**V**		**V**							
					**1**		**2**		**3**		**>=3**	
1 COVID-19 infection	50		1008		75		394		364		175	
No	38		717		49		266		269		133	
Unsure	6		143		6		58		59		20	
Long CoV still symptomatic	5	10.0%	89	8.8%	10	13.3%	47	11.9%	20	5.5%	12	6.9%
Transient Long Cov	1		59		10		23		16		10	
2 COVID 19 infections	21		574		57		231		217		69	
No	12		343		28		132		141		42	
Unsure	2		95		9		42		32		12	
Long CoV still symptomatic	4	19.0%	84	14.6%	15	26.3%	36	15.6%	26	12.0%	7	10.1%
Transient Long Cov	3		52		5		21		18		8	
3 COVID 19 infections	6		173		29		75		55		14	
No	2		78		11		37		23		7	
Unsure			30		6		13		9		2	
Long CoV still symptomatic	2	33.3%	43	24.9%	11	37.9%	15	20.0%	14	25.5%	3	21.4%
Transient Long Cov	2		22		1		10		9		2	

**ANWERS IN SPANISH**	**No V**		**V**	**OR**	**V**							
				**NoV vs V**	**1**		**2**		**3**		**>=3**	
1 COVID-19 infection	27		1519		68		297		251		130	
No	19		937		41		173		165		80	
Unsure	2		272		11		67		36		21	
Long CoV still symptomatic	2	7.4%	196	12.9%	11	16.2%	34	11.4%	31	12.4%	21	16.2%
Transient Long Cov	4		114		5		23		19		8	
2 COVID 19 infections	26		802		41		170		139		38	
No	15		409		19		80		75		23	
Unsure	4		162		4		34		32		9	
Long CoV still symptomatic	5	19.2%	171	21.3%	15	36.6%	45	26.9%	19	13.7%	4	10.5%
Transient Long Cov	2		60		3		11		13		2	
3 COVID 19 infections	7		273		26		43		44		20	
No	4		116		10		19		19		8	
Unsure			48		6		7		7		4	
Long CoV still symptomatic	2	28.6%	72	26.4%	7	26.9%	14	32.6%	9	20.5%	5	25.0%
Transient Long Cov	1		37		3		3		9		3	

Table S3. Number of Long COVID cases (LC) depending on the number of COVID-19 recorded infections (n infection) and the number of COVID-19 vaccines administered (nV), before (nV preinf) or after (nV postinf) the first COVID-19 infection.

	**n V**					***p***	**No V**	***P***
**n infection**	**n V preinf**	**1**	**2**	**3**	**>=3**	**1 vs ≥3 doses**		**NV vs ≥3 doses V preinf**
? (no date of infection)	LC/COV	7/5811 (0.12%)	13/21171 (0.06%)	10/12447 (0.08%)	15/21556 (0.07%)		21/75685 (0.03%)	
1	LC/COV	2/1342 (0.1%)	22/7272 (0.3%)	11/3778 (0.3%)	13/5992 (0.2%)		92/21608 (0.42%)	
OR vs 1 dose			2.03	1.95	1.45	0.61	1.96	0.02*
2	LC/COV	14/601 (2.3%)	12/934 (1.3%)	5/578 (0.9%)	12/835 (1.4%)		18/1833 (0.97%)	
OR vs 1 dose			0.56	0.38	0.62	0.22	0.69	0.30
>=3	LC/COV	2/70 (2.8%)	3/106 (1.9%)	2/87 (2.2%)	2/94 (2.1%)		10/136 (6.8%)	
OR vs 1 dose			0.67	0.81	0.75	0.77	3.29	(0.04)
**n infection**	**n V postinf**	**1**	**2**	**3**	**>=3**			
1	LC/COV	55/3854 (1.4%)	42/2523 (1.6%)	25/1314 (1.9%)	49/2193 (2.2%)			
OR vs 1 dose			1.16	1.32	1.53	0.02*		
2	LC/COV	4/113 (3.5%)	4/72 (5.6%)		5/103 (4.9%)			
OR vs 1 dose			1.57	47	1.37	0.64		
>=3	LC/COV	4/7 (57%)	7	1/5 (20%)	1/23 (4.3%)			
OR vs 1 dose				0.35	0.07	(0.04)		

*Statistical p values are calculated between 1 and >3 doses received and between non vaccinated and those full vaccinated receiving >=3 doses in patients with the same number of recorded infections. Significant differences are marked with * when at least 5 cases were detected per category.

Figure S1. Number of hospitalizations during the first 6 months of 2025.

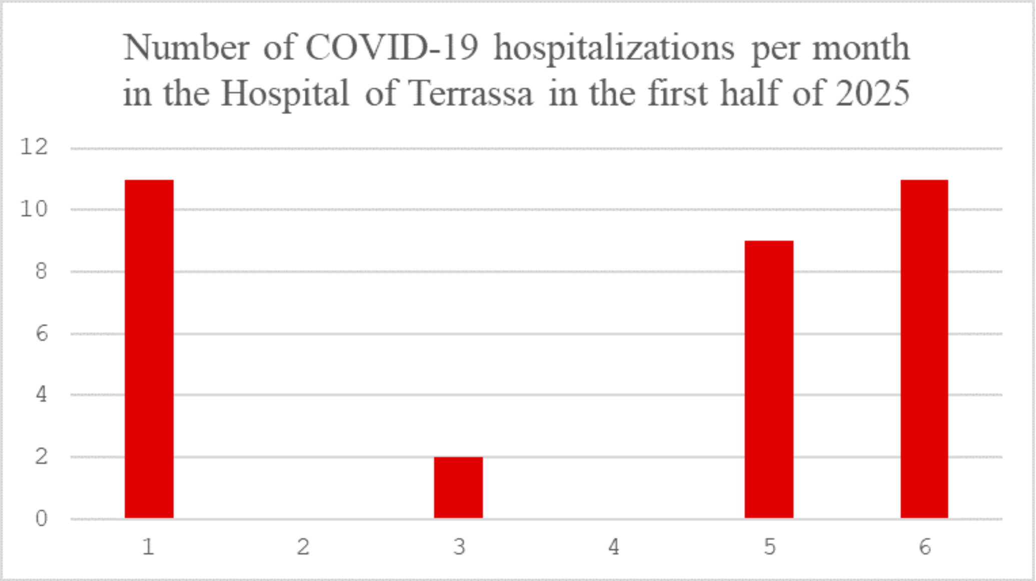

Supplement: Supplementary file 1 [file vaccines-13-00905-s001.zip › vaccines-3768551-supplementary R2.pdf]
